# Plant defences against ants provide a pathway to social parasitism in butterflies

**DOI:** 10.1098/rspb.2015.1111

**Published:** 2015-07-22

**Authors:** Dario Patricelli, Francesca Barbero, Andrea Occhipinti, Cinzia M. Bertea, Simona Bonelli, Luca P. Casacci, Simon A. Zebelo, Christoph Crocoll, Jonathan Gershenzon, Massimo E. Maffei, Jeremy A. Thomas, Emilio Balletto

**Affiliations:** 1 Zoology Unit, Department of Life Sciences and Systems Biology, University of Turin, Via Accademia Albertina 13, Turin 10123, Italy; 2 Plant Physiology Unit, Department Life Sciences and Systems Biology, University of Turin, Innovation Centre, Via Quarello 15/A, Turin 10135, Italy; 3 Department of Plant and Environmental Sciences, Faculty of Sciences, University of Copenhagen, Thorvaldsensvej 40, Frederiksberg C 1871, Denmark; 4 Max Planck Institute for Chemical Ecology, Beutenberg Campus, Hans-Knoell-Strasse 8, Jena 07745, Germany; 5 Department of Zoology, University of Oxford, The Tinbergen Building, South Parks Road, Oxford OX1 3PS, UK

**Keywords:** gene expression, plant volatiles, myrmecophily, host detection, ant, *Maculinea*

## Abstract

Understanding the chemical cues and gene expressions that mediate herbivore–host-plant and parasite–host interactions can elucidate the ecological costs and benefits accruing to different partners in tight-knit community modules, and may reveal unexpected complexities. We investigated the exploitation of sequential hosts by the phytophagous–predaceous butterfly *Maculinea arion*, whose larvae initially feed on *Origanum vulgare* flowerheads before switching to parasitize *Myrmica* ant colonies for their main period of growth. Gravid female butterflies were attracted to *Origanum* plants that emitted high levels of the monoterpenoid volatile carvacrol, a condition that occurred when ants disturbed their roots: we also found that *Origanum* expressed four genes involved in monoterpene formation when ants were present, accompanied by a significant induction of jasmonates. When exposed to carvacrol, *Myrmica* workers upregulated five genes whose products bind and detoxify this biocide, and their colonies were more tolerant of it than other common ant genera, consistent with an observed ability to occupy the competitor-free spaces surrounding *Origanum*. A cost is potential colony destruction by *Ma. arion*, which in turn may benefit infested *Origanum* plants by relieving their roots of further damage. Our results suggest a new pathway, whereby social parasites can detect successive resources by employing plant volatiles to simultaneously select their initial plant food and a suitable sequential host.

## 1. Introduction

A critical choice for many animals that eschew parental care is where and when to deposit their offspring. In most butterflies, this is seen in the females' use of visual, chemical [1] and microclimatic [2] cues to oviposit on small subsets of their host-plant(s) which possess attributes that optimize brood fitness [2,3]. For certain of the approximately 100 000 insect species [4,5], including perhaps a quarter of all butterflies, whose immature stages interact with ants (myrmecophiles), selection may also favour appropriate plants that harbour certain mutualistic ant species [6]. For the approximately 10% of myrmecophiles that infiltrate and exploit ant colonies as social parasites, egglaying near colonies of their host-ant taxon is in general obligatory [5,7].

**Figure 1.**
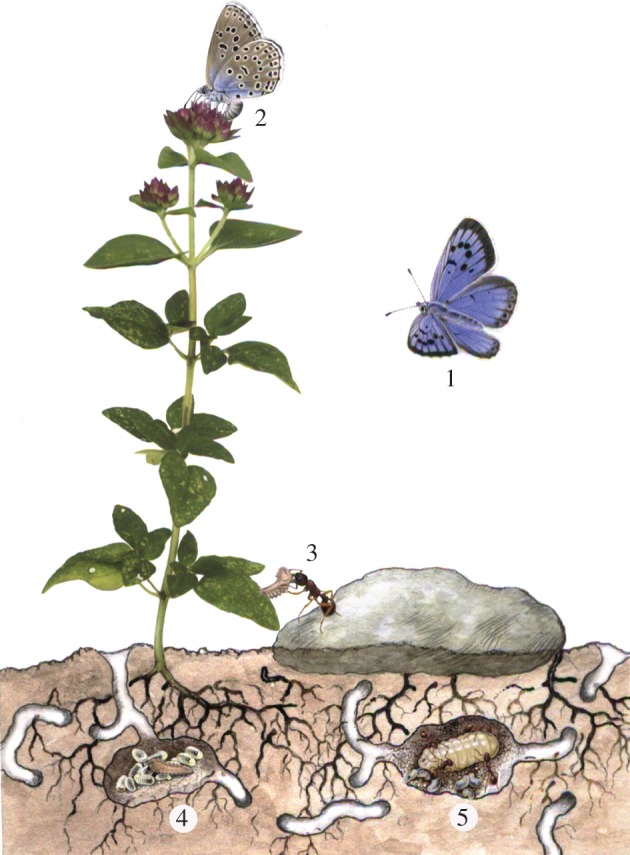
Life cycle of *Ma. arion* and its interaction with *Myrmica* ants and *O. vulgare*, indicated by this and previous [5,11–14] studies. (1) Female butterfly is attracted to flowers then, when close, by volatiles promoting oviposition (2) on flowerbuds of *O. vulgare* plants that co-occur with a nest of any *Myrmica* species; (3) final-instar larvae abandon *O. vulgare* and are adopted by *Myrmica* ants, in whose nests they live for 11 months, feeding on ant brood (4, 5) and acquiring more than 98% of their final biomass (5). Larval survival in the study race of *Ma. arion* is highest with *Myrmica scabrinodis* and *Myrmica sabuleti* and zero with ants of other genera [15].

Many insect social parasites have evolved from phytophagous ancestors [6,8,9] and are constrained, during early larval development, by an obligate need to feed on specific plant tissues before infiltrating ant colonies for an extended final instar during which most of their ultimate biomass is acquired ([8,10–12]; figure 1). With average realized fecundities seldom exceeding 50 eggs laid per wild female [5,11], this lifestyle poses the logistical challenge of locating sufficient proximate hosts for a population to persist. That constraint is shared by many true parasites that sequentially exploit more than one unrelated host species, but most overcome it by producing thousands of propagules, by switching host between generations rather than within the same one, and often by having a primary host that interacts trophically with a secondary one (the vector) [16,17].

What guides oviposition in insect social parasites with sequential hosts is unknown, despite a longstanding controversy exemplified by whether female *Maculinea* (large blue butterfly) species can detect initial host-plants growing close to colonies of their specific host species of *Myrmica* ant, and if so, whether they respond directly to ant cues or indirectly to growth forms of their foodplants that inhabit the same niche as that (or those) species of ant [13,18–21]. An obstacle to using *Myrmica* odours as a cue is that the known hosts in this ant genus seldom forage at times when the butterfly oviposits (10–16 h), the workers lay ephemeral trails and rarely climb plants, and the female butterfly exhibits no behaviour to detect ant nests [8,13] (by contrast, certain lycaenids that coexist with other ant genera that possess other attributes have indeed evolved the ability to detect their obligate ant mutualist or host prior to oviposition [6,8,22,23]). Nevertheless, we recently established that *Maculinea arion* females oviposit disproportionately often on *Origanum vulgare* plants situated near to a colony of *Myrmica* (of any species), and avoid other ant genera which their larvae cannot exploit [15]. It is well known that many plants synthesize deterrent volatile organic compounds (VOCs) in response to tissue damage by insects [24,25]. Here, we show that this social parasite can detect, and is attracted by, individual foodplants whose chemical profile has been modified by subterranean, non-trophic interactions with *Myrmica* ant nests.

## 2. Material and methods

### (a) Establishing terraria

*Myrmica sabuleti* and *Myrmica scabrinodis* colonies (primary host of *Ma. arion*, respectively, in west Europe and Italy) and *O. vulgare* seeds were collected in the Parco Fluviale Gesso e Stura, North Italy, where a previous study of *Ma. arion* oviposition behaviour had been made [15]. Twelve terraria (electronic supplementary material, figure S1) were established, each containing four *O. vulgare* plants grown from seedlings maintained at 26°C, 60% humidity and 120 µmol m^−2^ s^−1^ light under a 16 L : 8 D regime. Six also contained one *Myrmica* colony each, while six controls did not. *Myrmica* ants were fed on a standard diet of sugar and dead *Spodoptera littoralis* larvae [26].

### (b) Collection and analysis of plant volatiles

*Origanum vulgare* VOC emissions were assayed by stir-bar sorptive extraction (SBSE) [27] using bars placed in each terrarium. SBSEs were conditioned for 1 h at 250°C in a helium flow, then placed above a jar covering phenologically similar *O. vulgare* plants for 48 h (*n* = 41). After absorption, each stir bar was rinsed with double distilled water (Millipore, USA), dried and stored into a sterile vial at −20°C before analysis within 2 days by extraction using the Gerstel TDU Desorption Unit System and gas chromatography coupled to mass spectrometry (GC-MS). Quantification was performed by external calibration curve. Initially, separate assays (*n* = 20) were made of the impact of *My. sabuleti* and *My. scabrinodis* but as no difference was detected in their induced emissions, their data were pooled in later analyses. Further details of the collection and analysis of *O. vulgare* emissions are given in the electronic supplementary material.

### (c) Bioassays of responses of gravid female *Maculinea arion* to volatile organic compounds

Choice tests were performed on 10 gravid female butterflies per test in a 15 cm diameter Y-tube olfactometer housed in a blackened box with constant diffused fluorescent light shining directly above its centre. For 10 min, each female was offered a choice between two arms, each connected to a 4 l glass desiccator receiving a GC-grade constant air flow of 200 ml min^−1^. Stimuli were presented simultaneously in each trial and were alternated in the two arms between tests. A positive choice was registered only when a female entered and remained 1 min in one arm, rather than stayed in the initial tube or flew between both arms. Between tests, all glassware was carefully washed and air-dried at 120°C for 4 h. Flasks contained control or treated *O. vulgare* plants, while in other experiments synthetic thymol and carvacrol were applied to assess whether females could discriminate between these isomers. To test whether *Ma. arion* might use host-ant nest volatiles as an oviposition cue [18,20], we also used *My. scabrinodis* nest soil collected at our experimental site. As a control, we used the same volume of the same soil that had been sterilized by autoclaving to remove volatiles. In both cases, ants were removed from the soil before testing.

### (d) Electroantennogram measurements of gravid female *Maculinea arion*

Antennae of *Ma. arion* were excised at the base of the flagellum. The tip of the terminal segment was cut using micro-scissors, and the antennae (*n* = 8) were connected to an electrophysiology apparatus. The excised tip was placed on the antennal holder (two 1.7 mm diameter wells containing 0.1 M KCl); the recording electrode was connected to the base of each antenna with the reference electrode connected to its tip. Baseline responses of antennae were established from exposure to clean GC-grade air, and compared with responses when fluxing ant allomones, plant volatiles and synthetic pure compounds or mixtures. VOCs were fluxed towards the mounted antenna on the electroantennogram (EAG) apparatus with clean air as carrier gas. Synthetic carvacrol, thymol and Z-3-hexen-l-ol were obtained from Sigma-Aldrich (Milan, Italy) (10 µg per desiccator). VOCs from treated and untreated *O. vulgare* from 4 l glass jars (see above), synthetic mixtures and extracts from solitary ant nests were flowed. Electrical signals were pre-amplified and their intensity measured using a 0.052 V step input; the amplified output was 6.49 V. Responses were expressed as mean differences to the standard (Z-3-hexen-l-ol), Dimethyl sulfoxide (Sigma-Aldrich, USA) and clean air after exposing the antenna alternately, every 30 s, to a test chemical or to clean air.

### (e) Ant reactions to carvacrol

To assess whether significant differences of resistance rate occur upon acute exposure to carvacrol in the commonest ant species of the study site, two colonies each of five ant genera (*My. scabrinodis*, *Tetramorium caespitum*, *Lasius alienus*, *Tapinoma erraticum* and *Formica cinerea*) were kept for one week on standardized diets [26] before workers were exposed in sealed 6 cm Petri plates (representing a nest chamber) to an atmosphere containing 20 ppm carvacrol: survival was assessed as minutes before death. Parallel controls were tested using clean air but no ant died in the absence of carvacrol. At the end of the experiment, ants were frozen in liquid N_2_ and kept at −80°C for gene expression analyses.

### (f) Isolation of total RNA and gene expression analysis

#### (i) *Origanum vulgare* genes involved in terpene metabolism

To evaluate the effect of the presence of ants on terpenoid metabolism in *O. vulgare*, we analysed the expression level of terpene synthases and cytochrome P450s previously identified as being involved in the production of the main VOC components, carvacrol and thymol, in this plant species [28,29]. Full details about total RNA extraction, cDNA synthesis and gene expression analysis are given in the electronic supplementary material.

#### (ii) Ant genes involved in response to carvacrol

To understand the possible mechanism underlying the response to carvacrol exposure in different ant species, we analysed the expression level of known genes related to odour perception and xenobiotic detoxification in insects. Full details of the rationale used to choose candidate genes are given in the electronic supplementary material together with the methodologies used for total RNA extraction, cDNA synthesis and gene expression analysis.

### (g) Competitive advantage of *Myrmica* ant species to inhabit ground near *Origanum vulgare* plants

Two hundred and seventy pitfall traps were placed on our main study site, 190 within 10 cm of an *O. vulgare* plant [15] and 80 beneath vegetation of similar composition, sward and soil structure but situated more than 2 m from any *O. vulgare* plant.

### (h) Damage caused to *Origanum vulgare* by *Maculinea arion* larvae

The number of individual florets per flowerhead eaten by 13 *Ma. arion* larvae between hatching and the final moult before abandoning the foodplant was counted daily in the wild, together with the number of florets available per flowering plant spike, the number of flowering spikes per plant and the mean density of larvae surviving to final instar on a UK site that supported high densities of *Ma. arion*.

### (i) Statistics

Kolmogorov–Smirnov tests were used to assess the data distribution type. Accordingly, parametric or non-parametric analysis of variance was used to assess differences in VOC amounts, although for consistency just one type of test is presented in each table. Choice data were analysed using *χ*^2^ tests. Kruskal–Wallis analysis was used to test for differences in mean ant survival times; pairwise differences between species were also calculated using Bonferroni correction, and Benjamini–Hochberg procedure was used to control for false discovery rate (FDR) in multiple tests. For genomic (log-transformed data) and chemical analyses, the overall datasets were expressed as mean values of at least three biological replicates, each one repeated (technical replicates). Significance of differences observed in datasets was tested by ANOVA and pairwise comparisons were assessed with Bonferroni post hoc. All statistical analyses were performed using SPSS (v. 18.0, Chicago). Other tests are indicated in the text.

## 3. Results

### (a) Response of *Origanum* plants to root disturbance by *Myrmica* ants

*Origanum vulgare* grown in the presence of *My. sabuleti* or *My. scabrinodis* had the same headspace concentrations of 20 major VOCs, including carvacrol (2-methyl-5-(1-methylethyl)-phenol) and thymol (electronic supplementary material, table S1; *p* = 0.990 and 0.583, respectively): we therefore combined data from these two congeners in subsequent analyses. Compared with the controls, *O. vulgare* grown in the presence of a *Myrmica* species had a headspace concentration of carvacrol that was nearly twice that of plants grown without ants nesting around and disturbing their roots (*p* = 0.00032, table 1). This aromatic oxygenated monoterpene is well known for its antifungal and insecticidal properties [30,31]. By contrast, the headspace concentration of the 19 other major volatiles did not change apart from that of the carvacrol precursor, γ-terpinene (table 1). A similar pattern was obtained for leaf extracts from wild plants growing in the field, except that carvacrol's isomer thymol was also significantly elevated in *O. vulgare* growing near *Myrmica* colonies (*p* < 0.0001; electronic supplementary material, table S2).

**Table 1.** The abundance of major compounds found in the headspace of *O. vulgare* grown in terraria with or without a *Myrmica* ant colony. (Mean values between treatment (*n* = 20) and control plants (*n* = 21) were compared by Kruskall–Wallis tests (*K*). Data are expressed as μg g^−1^ of fresh weight ± s.e.m. after 48 h of sorption with SBSE (see Material and methods). Asterisks (*) denote significant *p*-values controlled for FDR by using the Benjamini–Hochberg procedure.)

compound	Kovats index	plants with *Myrmica*	control plants without *Myrmica*	*K*	*p*-value
*p*-cymene	1024	0.912 ± 0.209	0.916 ± 0.198	0.083	0.773
γ-terpinene	1055	0.924 ± 0.185	0.804 ± 0.171	22.286	0.000002*
*trans*-sabinene hydrate	1098	1.016 ± 0.242	0.923 ± 0.240	2.871	0.090
methylthymyl ether	1173	1.064 ± 0.284	1.067 ± 0.255	0.062	0.804
thymol	1290	1.290 ± 0.311	1.911 ± 0.439	1.088	0.297
carvacrol	1299	1.310 ± 0.291	0.892 ± 0.202	12.957	0.000319*
α-cubebene	1351	0.867 ± 0.193	0.833 ± 0.180	1.046	0.307
α-copaene	1377	1.040 ± 0.219	0.986 ± 0.195	0.497	0.481
β-ylangene	1421	4.499 ± 0.854	4.309 ± 0.644	0.011	0.917
*γ*-elemene	1437	1.327 ± 0.280	1.287 ± 0.261	0.098	0.754
α-bergamotene	1413	0.831 ± 0.198	0.824 ± 0.182	0.393	0.531
γ-muurolene	1480	1.960 ± 0.370	1.665 ± 0.261	1.035	0.309
bicyclosesquiphellandrene	1484	1.282 ± 0.251	1.175 ± 0.213	0.787	0.375
germacrene-d	1485	5.722 ± 1.128	5.514 ± 0.784	0.006	0.938
α-amorphene	1485	1.052 ± 0.251	0.960 ± 0.219	2.532	0.112
(*E*)-β-farnesene	1457	4.638 ± 0.854	4.389 ± 0.909	0.197	0.657
bicyclogermacrene	1500	1.056 ± 0.216	0.960 ± 0.186	1.503	0.220
α-bisabolene	1507	0.827 ± 0.211	0.832 ± 0.199	0.340	0.560
δ-cadinene	1523	0.941 ± 0.211	0.969 ± 0.206	0.360	0.548
α-humulene epoxide	1608	1.786 ± 0.380	1.208 ± 0.264	2.451	0.117

**Figure 2.**
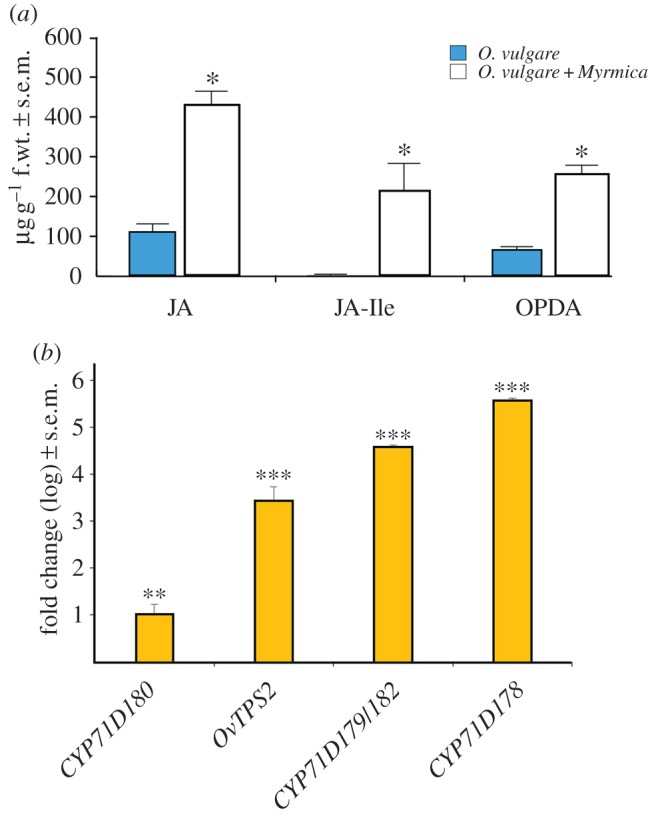
Effects of *Myrmica* ants on *O. vulgare* defence hormone production and gene induction. (*a*) *Myrmica* presence induces increases in the defence hormone JA, its conjugate JA-Ile and JA precursor OPDA; f.wt = fresh weight; *p < 0.001 between plants with and without *Myrmica*. (*b*) *Myrmica* presence causes a 10-fold induction for a monoterpene synthase gene (*OvTPS2*) that encodes a protein catalysing the formation of γ-terpinene, a direct precursor for thymol and carvacrol. Large increases also occurred in transcript levels of three cytochrome P450 genes involved in carvacrol and thymol biosynthesis: *CYP71D180*, *CYP71D179/182* and *CYP71D178*; **p < 0.01, ***p < 0.001.

We next measured the levels of certain plant hormones and genes associated with monoterpene biosynthesis. In leaves of *O. vulgare* plants interacting with *Myrmica* colonies (figure 2*a*), there was a significant increase in the defence hormone jasmonic acid (JA) (ANOVA, *F*_1,5_ = 62.32, *p* = 0.001), its conjugate (*3R*,*7S*)-jasmonoyl-l-isoleucine (JA-Ile) (ANOVA, *F*_1,5_ = 141.625, *p* < 0.001) and the JA precursor, 12-oxo phytodienoic acid (OPDA) (ANOVA, *F*_1,5_ = 244.905, *p* < 0.001) [32]. There was also a 10-fold induction in the presence of *Myrmica* (figure 2*b*) for a monoterpene synthase gene (*OvTPS2*) (ANOVA, *F*_1,5_ = 273.055, *p* = 0.0008) that encodes a protein catalysing the formation of γ-terpinene, a direct precursor for thymol and carvacrol [28,33], as well as large increases in transcript levels of three cytochrome P450 genes involved in carvacrol and thymol biosynthesis: *CYP71D180* (ANOVA, *F*_1,5_ = 48.913, *p* = 0.002), *CYP71D179/182* (ANOVA, *F*_1,5_ = 3388.328, *p* < 0.00001) and *CYP71D178* (ANOVA, *F*_1,5_ = 6919.464, *p* < 0.00001). By contrast, the expression levels of genes involved in biosynthesis of other volatile terpenes (electronic supplementary material, table S3) showed no significant differences between ant-treated and control plants.

These results suggested that *Myrmica* ant colonies may stimulate *O. vulgare* plants, leading to elevated levels of the defence hormones JA and JA-Ile, and subsequently to increases in monoterpene biosynthesis via upregulation of selected gene transcripts [32].

### (b) Effect of carvacrol emissions on *Maculinea arion* behaviour

**Figure 3.**
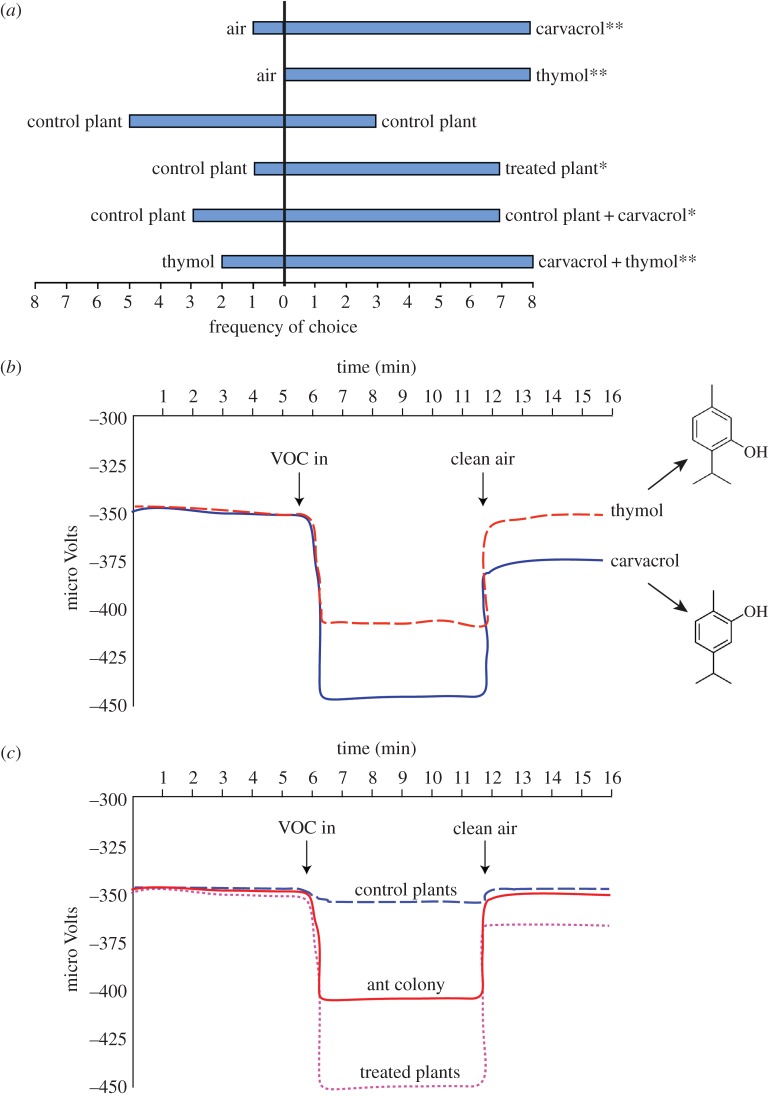
Responses of *Ma. arion* to ant-induced plant volatiles. (*a*) Olfactometry choice bioassays of gravid female *Ma. arion* show a strong preference for carvacrol and its isomer thymol over clean air, as well as for the odour of *O. vulgare* exposed to ants (=treated plant) or of controls dosed with carvacrol rather than that of isolated control plants. Females also chose a blend of carvacrol and thymol rather than thymol alone; *p < 0.05, **p < 0.01. (*b*) EAG of *Ma. arion* antennal responses to the two main aromatic monoterpenes emitted by *O. vulgare*. (*c*) Antennal responses in EAGs to odours from plants grown with *Myrmica* among their roots (=treated) and to the odour of *My. scabrinodis* ant colonies.

Having demonstrated that *O. vulgare* plants in the optimum phenological state for *Ma. arion* oviposition [13] synthesize and emit elevated levels of aromatic oxygenated monoterpenes when *Myrmica* ants are present, we tested whether carvacrol, or its isomer thymol, affect *Ma. arion* behaviour. In the bioassays performed with a Y-tube olfactometer (figure 3*a*), gravid female butterflies selected tubes containing carvacrol (*χ*^2^_2_ = 9.8, *p* = 0.007) or thymol (*χ*^2^_2_ = 10.4, *p* = 0.005) significantly more often than pure air, and responded more frequently to a blend of carvacrol and thymol than to pure thymol (*χ*^2^_2_ = 10.4, *p* = 0.005). Furthermore, females selected *O. vulgare* grown without a *Myrmica* colony to which synthetic carvacrol had been applied in preference to untreated *O. vulgare* plants (*χ*^2^_2_ = 7.4, *p* = 0.024) and showed a strong preference for the odour from plants grown in terraria containing ants compared with controls (*χ*^2^_2_ = 6.2, *p* = 0.045). By contrast, females displayed no attraction to the odours of soil taken from a host *Myrmica* nest, or for sterilized soil, but remained in the main portion of the Y-tube or flew randomly between the two branches.

Finally, in EAG experiments, isolated antennae of *Ma. arion* females generated distinct electrophysiological pulses when exposed to air containing thymol and, especially, carvacrol compared with clean air (figure 3*b*) (ANOVA, *F*_7,1110_ = 445.829, *p* < 0.001, Tukey test_Carvacrol versus Thymol_, mean difference = 14.45, *p* < 0.001). Antennae also responded much more strongly (figure 3*c*) to the induced VOCs of *O. vulgare* than to volatiles extracted directly from a *Myrmica* colony (Tukey test*_O. vulgare-_*_treated-with-ants versus *Myrmica*_, mean difference = 32.38, *p* < 0.001), although they did show a weak hyperpolarization when exposed to the latter (Tukey test*_Myrmica_*_versus control_ *_O. vulgare_*, mean difference = 13.70, *p* < 0.001).

### (c) Impact of carvacrol synthesis on *Myrmica* and other genera of ants

**Figure 4.**
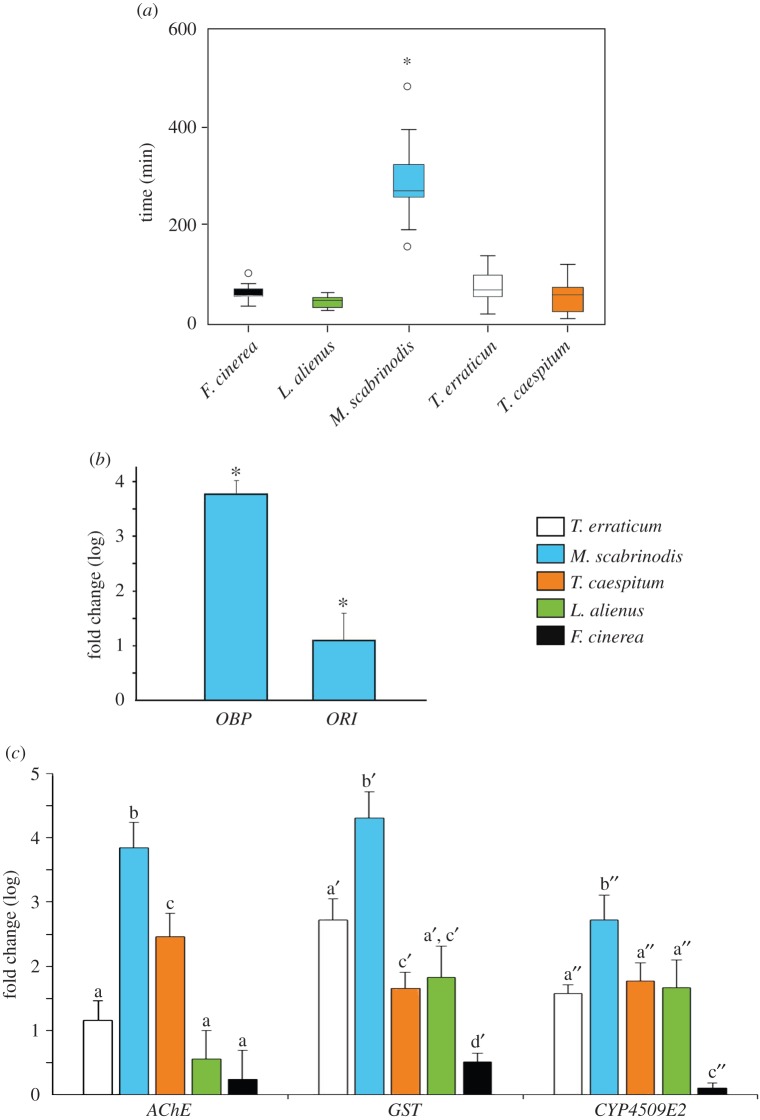
Tolerance of different ant genera to carvacrol. (*a***)** Worker survival times when entrapped in cells containing 20 ppm pure carvacrol. Boxplots show median, quartile, maximum and minimum survival; outliers are open circles; Kruskal–Wallis test_all ants_ H_4_ = 37.71, *n* = 63, d.f. = 4_,_ *p* < 0.001; pairwise: *My. scabrinodis* ≠ *L. alienus* (*U* = 41.577, *p* < 0.001), ≠ *T. caespitum* (*U* = 33.154, *p* < 0.001), ≠ *F. cinerea* (*U* = 27.077, *p* = 0.002), ≠ *T. erraticum* (*U* = 22.864, *p* = 0.023). (*b*) Gene expression of odourant receptor 1 and odourant-binding protein (*OBP*6) in *My. scabrinodis* ants after exposure to carvacrol. (*c*) Expression of detoxification-related genes in the five ant genera tested after exposure to carvacrol: acetylcholinesterase (*AChE*), *GST* and a cytochrome P450 (*CYP4509E2*). Error bars indicate s.e.m. for each gene, different letters indicate significant (*p* < 0.05) differences.

We had previously observed that *Ma. arion* eggs are laid disproportionately often on *O. vulgare* plants growing near the nests of *Myrmica* species in the wild [15], yet an attraction to carvacrol is unlikely to benefit *Ma. arion* if damage from non-host-ant genera induces similar emissions. Knowing that carvacrol is a biocide for many insects [30,31], we tested its impact on the commonest ant genera on our *Ma. arion* sites by confining their workers in closed cells containing 20 ppm of this volatile. In this atmosphere, *My. scabrinodis* survived four to six times longer than workers from other genera (figure 4*a*). Moreover, gene expression of the *My. scabrinodis* odourant-binding protein *OBP6* (ANOVA, *F*_1,5_ = 619.095, *p* < 0.0001) and odourant receptor 1 (*OR1*) (ANOVA, *F*_1,5_ = 12.659, *p* = 0.024) showed a significant upregulation in workers exposed to carvacrol (figure 4*b*), suggesting *Myrmica*'s ability to react to the compound at the molecular level. Furthermore, three detoxification-related genes were significantly more upregulated in *My. scabrinodis* compared with other ant genera (figure 4*c*) upon exposure to carvacrol: acetylcholinesterase (*AChE*; ANOVA, *F*_4,14_ = 43.04, *p* < 0.00001), which is responsible for the hydrolysis of acetylcholine at synaptic regions of cholinergic nerve endings in the ant central nervous system [34]; glutathione *S* transferase (*GST*; ANOVA, *F*_4,14_ = 51.55, *p* < 0.00001), a primary defence against xenobiotics [35]; and a cytochrome P450 (*CYP4509E2*; ANOVA, *F*_4,14_ = 29.42, *p* < 0.00001), known to metabolize synthetic chemicals (insecticides/pesticides) and host-plant allelochemicals [36].

Although workers inhabiting natural nests move freely between nest chambers and above-ground where concentrations may be lower, we tested ant responses to 20 ppm of carvacrol (=3.4 times higher than that emitted into *Origanum* headspace but one-third of the induced concentration within leaf tissues) to compare differences in species' tolerance to acute exposure to this biocide (figure 4*a*). Taken together, our data suggest that, while *O. vulgare* may produce carvacrol as an insect repellent, *My. scabrinodis* possesses a higher capability to detoxify this volatile than ant species from other genera, and an inducible ability to recognize and bind the molecule.

### (d) Benefits and costs

For *Myrmica* species, the apparent benefit of their relative tolerance to carvacrol is an ability to inhabit the competitor-free space surrounding *O. vulgare* plants (table 2; *Myrmica* versus non*-Myrmica*, *χ*^2^_1_ = 52.0, *p* < 0.001). A cost is parasitism in the few sites occupied by *Ma. arion* (an International Union for Conservation of Nature-listed rarity [11]), as infection by the butterfly typically destroys the host species' colony or causes it to desert, owing to the often total loss of brood to parasitic caterpillars that acquire more than 98% of their ultimate biomass from devouring ant larvae [14]. From the plant's perspective, the attraction of gravid *Ma. arion* females has a much smaller negative impact as an individual caterpillar eats approximately 17 florets before abandoning its foodplant, representing 2% of seed production of the individual plants supporting caterpillars on high-density *Ma. arion* sites (table 3).

**Table 2.** The distribution of *Myrmica* species and other (non-*Myrmica*) genera of ants beneath *O. vulgare* plants and in equivalent habitat more than 2 m away from *O. vulgare.*

habitat patches	*N*	*Myrmica* species present	non-*Myrmica* ant genera present	no ants
beneath *Origanum*	190	139 (73%)	18 (9%)	36 (19%)
more than 2 m from *Origanum*	80	36 (45%)	44 (55%)	0

**Table 3.** The impact of *Ma. arion* larvae on seed production by *O. vulgare* plants on which females laid one or more eggs. (Sampling was from the site with the highest density of *Ma. arion* known in Europe; mean values are given ±s.e.m.)

density of *Ma. arion* larvae per occupied plant	no. florets eaten per *Ma. arion* larva	no. florets per flowering spike of occupied plants	no. flowering spikes per plant	% florets eaten by *Ma. arion* on occupied plants
1.45 ± 0.1	17.08 ± 0.6	310 ± 36	5.23 ± 0.53	2.02 ± 0.31

## 4. Discussion

We have yet to determine how a colony of omnivorous *Myrmica* ants causes sufficient damage to trigger increased defence hormones and monoterpene biosynthesis in *O. vulgare*, but a likely cause is their cutting of roots and rootlets to excavate cells. Although the specialized domestic root-aphids nurtured by *Myrmica* workers [37] might amplify plant defences in the field, these aphids were excluded from our laboratory experiments. Elsewhere in Europe, *Ma. arion* oviposits on flowers of the genus *Thymus*, a close relative of *O. vulgare. Thymus* species also emit thymol and carvacrol when damaged [38], and *Ma. arion* presumably employs the same cues in its interaction with *O. vulgare* or *Thymus* plants and *Myrmica* ant species throughout its range.

These results help to resolve a controversy as to whether female *Maculinea* butterflies, prior to oviposition, could select initial foodplants that coexist with their specific host species of *Myrmica* ant by using ant semio-chemicals as a cue [18–21], or whether they simply choose distinctive growth forms of plant that inhabit a similar niche to that of their host ant [13]. We show that neither hypothesis is correct, at least in the case of *Ma. arion*, and that although the butterfly antenna can respond weakly (in comparison to *Origanum* VOCs) to *Myrmica* odours, these exert no attraction for gravid females (figure 3). We therefore suggest that, after initially fluttering around pink flowers, the butterfly responds to plant VOCs induced by the presence of nests of various species in the genus *Myrmica* living in close proximity to *Origanum* plants, before further biasing egg distribution to a second subset of *Origanum* plants whose growth-form occupies a similar niche to that of the host species of *Myrmica* [13].

We consider that the described interactions are adaptive for all members of the *Ma. arion* community module (figure 1). An ability, when ovipositing, indirectly to detect the presence of the genus *Myrmica* benefits the butterfly because, on sites capable of supporting its populations, the large majority of *Myrmica* nests that coexist with the foodplant will belong to the primary host species [5,11]. Moreover, for the minority of eggs laid near a less appropriate congener species, there is a small (approx. 80% lower) chance of survival compared with none for individuals placed near other genera of ants [11,14]. For *Myrmica* ants, the highly localized cost of colony destruction by *Ma. arion* is presumably outweighed by an ability to dominate competitor-free spaces surrounding *Origanum*. While for the plant, the minor loss of seed to *Ma. arion* is presumably outweighed by the subsequent removal of any *Myrmica* colony damaging its roots and the cost of continuing to synthesize carvacrol. However, other aspects of these interactions remain unquantified, for example the precision with which carvacrol synthesis signals the proximity of a *Myrmica* colony to *Origanum*, or whether carvacrol is emitted more generally in response to insect herbivores. On current evidence, the latter seems unlikely, as herbivory is not cited as a cause in other studies of carvacrol synthesis, nor did we detect it when studying *Origanum* responses to the leaf-eating herbivore *S. littoralis* (A. Occhipinti 2014, personal communication). In addition, we have no data on whether *Ma. arion*'s early larval instars are exposed to carvacrol within *Origanum* flowerheads, and if so whether—like *Myrmica* (figure 4)—they can detect, bind and detoxify it.

More broadly, these results suggest that *Ma. arion* resembles many true endo-parasites in specializing on sequential hosts that interact closely with each other. It differs, however, in exploiting a non-trophic interaction that apparently emits so precise a signal that a typical butterfly, with an average realized fecundity of just 50 eggs per female, can nevertheless place sufficient eggs within the approximately 6 m^2^ territory of a suitable *Myrmica* colony for her offspring to experience a positive rate of intrinsic population growth [11]. We suspect that it may have been the ability to detect this, or comparable plant–ant interactions, that enabled the *Maculinea*, and others among multiple independent lines of phytophagous insect myrmecophiles [5,6,8], to evolve from mutualistic ancestors to enjoy the rewards of social parasitism (abundant food in an enemy-free space for the main period of growth) while retaining their ancestral traits of low fecundity [11] and possession of vulnerable phytophagous early young stages that are ill-adapted [9,12] to penetrate the formidable inner defences of ant host societies [37].

Although it is well known that plant volatile compounds provide reliable and easily detectable cues both in host-plant selection by insect herbivores [39] and in the attraction of herbivore enemies to herbivore-damaged plants [24,25], the *Maculinea*–*Origanum*–*Myrmica* interaction is the first example, to our knowledge, in which the same volatile signal directs both trophic and parasitic behaviours. The plant monoterpene carvacrol acts as an allomone with respect to *Myrmica* ants, while simultaneously attracting butterflies as a kairomone [25]. In this way, the butterfly chooses its initial host-plant not only for its intrinsic qualities as food for its larvae, but also for propinquity to its subsequent animal resource. These results suggest a new pathway and mechanism, whereby social and other parasites that possess sequential hosts can detect and exploit their successive resources.

## Data accessibility

All raw data concerning bioassays, other experiments, chemical analysis and gene expression are lodged with the Dryad database, file: DATASET_Patricelli_Barbero *et al.* http://dx.doi.org/10.5061/dryad.p14jc.
